# Down-regulation of miR-27a might inhibit proliferation and drug resistance of gastric cancer cells

**DOI:** 10.1186/1756-9966-30-55

**Published:** 2011-05-13

**Authors:** Xiaohong Zhao, Li Yang, Jianguo Hu

**Affiliations:** 1 Institute of Digestive Disease, Subsidary Hospital, The Medical University of Ningxia Province, Yin'chuan, 750001, Ningxia Province, China

**Keywords:** miR-27a, drug resistance, porliferation, gastric carcinoma

## Abstract

**Aims:**

Here we aimed to firstly investigate the role of miR-27a in proliferation and multidrug resistance of gastric cancer cells.

**Methods:**

The role of miR-27a in gastric cancer cells was detected using MTT assay, soft agar assay, flow cytometry assay, nude mice assay, real-time PCR, western blot and reporter gene assay, etc.

**Results:**

Down-regulation of miR-27a could inhibit porliferation of gastric cancer cells in vitro and in vivo. Down-regulation of miR-27a could also confer sensitivity of drugs on gastric cancer cells, and might increase accumulation and decrease releasing amount of adriamycin in gastric cancer cells. Down-regulation of miR-27a could significantly decrease the expression of P-glycoprotein and the transcriptional activity of cyclin D1, and up-regulate the expression of p21.

**Conclusions:**

MiR-27a might play important roles in porliferation and drug resistance of gastric cancer. MiR-27a might be considered as a useful target for cancer therapy.

## Introduction

Gastric cancer was one of the major causes of mortality in the world, especially in Asian countries. So far, the pathogenic mechanism underlying gastric carcinogenesis was not fully elucidated.

MicroRNAs (miRNAs) were a class of 22-nucleotide noncoding RNAs, which might function as regulators of gene expression [1]. More and more evidences showed that miRNAs might play important roles in various biological processes, including cell proliferation, apoptosis, tumorigenesis and MDR of cancer [2]. So far, the functions of gastric cancer related miRNAs were not clear. MiR-27a might mediate drug resistance of esophageal cancer cells through regulation of MDR1 and apoptosis [3]. However, the role of miR-27a in gastric cancer was not reported yet. To our knowledge, here we have firstly investigated the role of miR-27a in proliferation and multidrug resistance of gastric cancer cells.

## Materials and methods

### Cell culture

Human gastric cancer cell line, MKN45, was routinely maintained in DMEM medium (GIBCO, Carlsbad, CA, USA) supplemented with 10% fetal bovine serum at 37°C in humidified air containing 5% carbon dioxide air atmosphere.

### MiRNA transfection

Cells were plated in plates and cultured for 16 h, and then transfected with the antagomirs of miR-27a or control RNA (Lafayette, CO) as described previously [3].

### Real-time PCR

Total RNAs from cells were extracted and cDNA synthesis and amplification were performed as described previously [4]. Primers were designed as: MDR1, forward: 5'-CCCATCATTGCAATAGCAGG-3', reverse: 5'-TGTTCAAACTTCTGCTCCTGA-3'; cyclinD1, forward: 5'-GGAGCTGCTCCTGGTGAACA-3', reverse: 5'-TGTTGGGGCTCCTCAG GTTCA-3'; P21, forward: 5'-CCCGTGAGCGATGGAACT-3', reverse: 5'-CGAGGCACAAGGG TACAAGA-3'; P27, forward: 5'-CAAGTACGAGTGGCAAGAGG-3', reverse: 5'-GTAGAA GAATCGTCGGTTGC-3'. Comparative real-time PCR was performed in triplicate, including no-template controls. Relative expression was calculated using the comparative Ct method.

### Cell growth assay

Cells were seeded on a 96-well plate at 3 × 10^4 ^cells/well. Each sample has four replicates. Viable cells were counted by the MTT assay after 2, 4, 6, and 8 days.

### Soft agar assay

Soft agar assay was performed as described previously [5]. Each assay was performed in triplicate.

### Tumor growth in nude mice

Female athymic *nu/nu *mice, 5-6 weeks of age, were used in the experiment. The cells were resuspended in D'Hanks solution, and 5 × 10^6 ^cells in 0.2 ml were injected subcutaneously into the right flank of 4-week-age mice. Experimental and control groups had at least 6 mice each. Tumors were measured twice weekly, and the tumor volume was calculated.

### In vitro drug sensitivity assay

Vincristine (VCR), adriamycin (ADR), cisplatin (CDDP) and 5-fludrouracil (5-flu) were prepared before experiment. Drug sensitivity was evaluated using MTT assay as described previously [3].

### Flow cytometry assay (FCM)

Fluorescence intensity of intracellular ADR was detected by FCM as described previously [3].

### Western blot

Cellular proteins were separated on SDS-PAGE gels, and western blot was performed as described previously [3].

### Reporter gene assay

The pGL3-cyclin D1 vector and the control vector were prepared as described previously [3]. Briefly, 0.4 μg of reporter gene constructs was transfected into MKN45 cells using LipofectAMINE (Invitrogen) reagent according to the manufacturer's protocol. This transfection was done concurrently with the transfection of the antagomirs of miR-27a. Cells co-transfected with scrambled antago-miR-NC served as controls.

### Statistical analysis

All the data were presented as the mean ± SD. The significance of differences was determined with Student's t test or the χ2 test. *P *< 0.05 was considered statistically significant.

## Results

### Down-regulation of miR-27a inhibited the growth and tumorigenecity of gastric cancer cells

**Figure 1:**
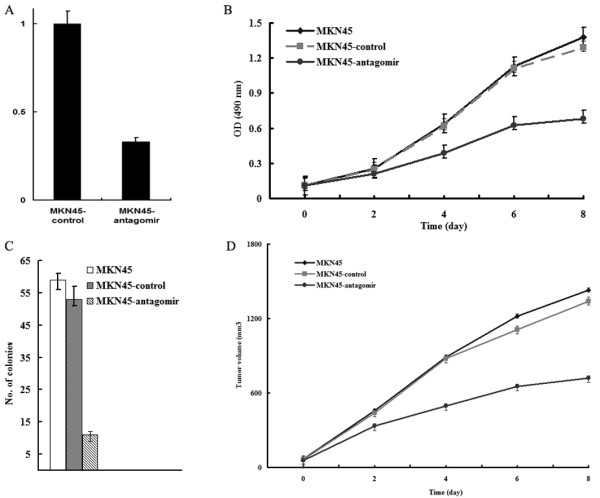
**ZNRD1 suppressed growth of gastric cancer cells in vitro and in vivo**. The data represented the mean ± SD of three independent experiments. A, Relative level of miR-27a in MKN45 cells after transfection. The mRNA level of the control cell (MKN45-control) was arbitrarily set at 1, and the mRNA levels of miR-27a in MKN45-antagomir cells were normalized to the control.B, the growth rate of the cells was detected using MTT assay. C, colony numbers of the cells were detected in soft agar. D, tumorigenicity of the cells in BALB/c nu/nu mice was detected. The volumes of tumors were monitored at the indicated time.

As Figure 1A showed, MKN45 cells were transfected with either the antagomirs of miR-27a or control RNA. The antagomirs of miR-27a could significantly inhibit the expression of miR-27a by almost 67% as compared with that of control. Cell growth was assayed, and down-regulation of miR-27a significantly inhibited proliferation of MKN45 cells as compared with control (P < 0.05) (Figure 1B). MKN45 cells and their transfectants were seeded in soft agar and colon formation was assessed. As shown in Figure 1C, down-regulation of miR-27a significantly inhibited the number of colonies formed by gastric cancer cells. Tumorigenesis was found profoundly decreased in miR-27a-downregulating cells as compared with control groups (Figure 1D), suggesting that down-regulation of miR-27a might inhibit the growth of MKN45 cells *in vitro *and *in vivo*.

### Down-regulation of miR-27a might reverse drug resistance of gastric cancer cells

**Figure 2:**
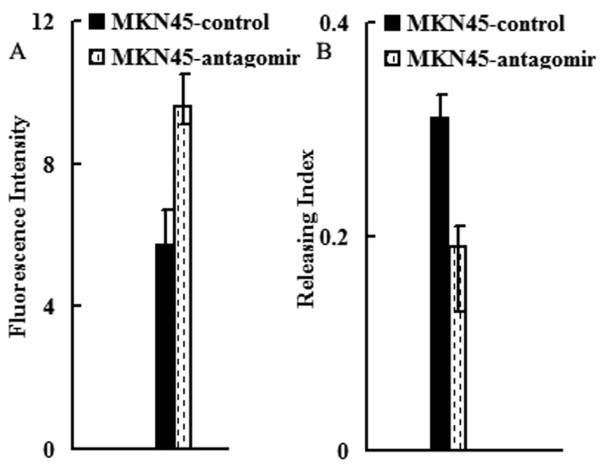
**Effect of miR-27a on ADR intracellular accumulation and releasing of MKN45 cells**. A, Fluorescence intensity analysis of intracellular ADR in cells; B, ADR releasing index of cells.

As shown in Table 1, the IC50 values of miR-27a antagomir cells for VCR, ADR and 5-flu were significantly decreased as compared with control cells. The ADR intracellular accumulation and releasing were explored using FCM assay. As shown in Figure 2A, B, increased accumulation and decreased releasing index of ADR of miR-27a antagomir cells was observed as compared with control cells (p < 0.05).

**Table 1:** IC50 values (μg/mL) of drugs for gastric cancer cells

	VCR	ADR	5-Flu	CDDP
MKN45	6.12 ± 0.22	6.41 ± 0.15	5.24 ± 0.11	5.11 ± 0.13
MKN45-control	5.81 ± 0.16	6.22 ± 0.11	4.88 ± 0.15	4.38 ± 0.26
MKN45-antagomir	1.68 ± 0.11 ^a^	1.93 ± 0.12 ^a^	1.79 ± 0.08 ^a^	1.16 ± 0.07 ^a^

Data were represented as mean ± SD of 3 independent experiments. ^a ^*p *< 0.05 *vs *MKN45 and MKN45-control cells.

### Effect of mir-27a on protein regulating proliferation and drug resistance

**Figure 3:**
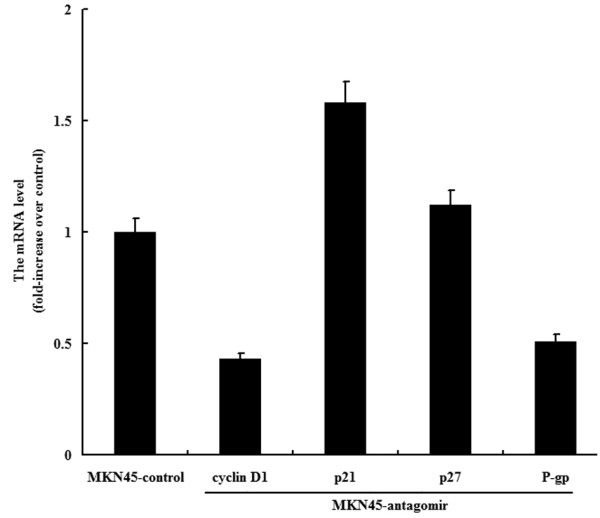
**Effects of a miR-27a on expression of cyclin D1, P-gp, p21 and p27 in gastric cancer cells**. The mRNA level of the samples treated with a control RNA was arbitrarily set at 1, and the genes' mRNA levels of the transfected cells were normalized to the control.

**Figure 4:**
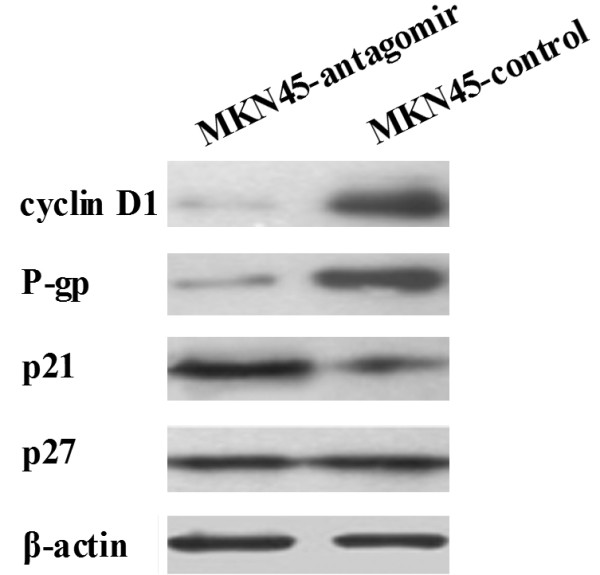
**Western blot analysis of cyclin D1, P-gp, p21 and p27 in gastric cancer cells**. β-actin was used as an internal control.

**Figure 5:**
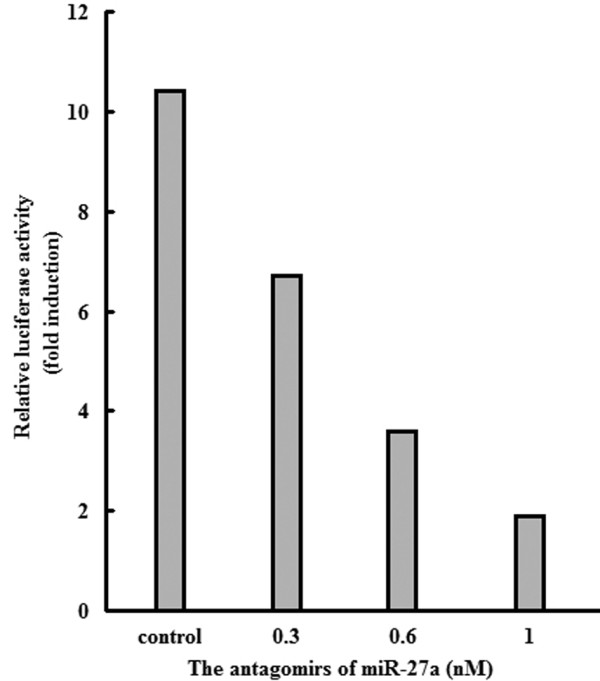
**The effect of antagomirs of miR-27a on cyclin D1 promoter activity**. Luciferase reporter assay was detected by cotransfection of this reporter gene (0.2 μg/well) with increasing amounts of antagomirs of miR-27a (0.3, 0.6, and 1 nM) in MKN45 cells. Cells co-transfected with scrambled antago-miR-NC served as controls.

The expression of P-glycoprotein, cyclin D1, p21 and p27 was detected in the gastric cancer cells using real-time PCR (Figure 3) and western blot (Figure 4). Down-regulation of miR-27a could significantly decrease the expression of P-glycoprotein and cyclin D1, and up-regulate the expression of p21. To evaluate whether cyclin D1 was a genuine target of miR-27a, luciferase reporter assay was performed. As shown in Figure 5, co-transfection of increasing amounts of antagomirs of miR-27a with cyclin D1 reporter gene led to significantly decrease in cyclin D1 promoter activity, suggesting that miR-27a might target cyclin D1.

## Discussion

Aberrant miRNA expression patterns had been described in a variety of malignancies. MiRNAs might play important roles in multiple developmental processes. MiR-27a was widely expressed in cancer cells and might function as an oncogene through regulating cell survival and angiogenesis [6-11]. In this study, we have firstly found that miR-27a might play important roles in mediating proliferation and drug resistance of gastric cancer.

To obtain a better model in which cells of the same origin could be compared, we transfected MKN45 cells with the antagomirs of miR-27a or control RNA. The results of MTT assay and soft agar assay revealed that down-regulation of miR-27a inhibited cell growth of gastric cancer cells in vitro, which was consistent with the data of nude mice assay. The study was aimed at investigating the effect of miR-27a on gastric cancer cells and more importantly, examining the mechanisms governing these effects. Here we clearly showed for the first time that miR-27a might mediate cell proliferation by regulation of cyclin D1 and p21. Cyclin D1 might play important roles in facilitating the transition from G_1 _phase into S. The results of luciferase reporter assay suggested that miR-27a might be a transcriptional regulator of the cyclin D1 gene.

The results of MTT assay indicated that down-regulation of miR-27a promoted drug sensitivity of gastric cancer cells. ADR was then used as probe to evaluate drug accumulation and retention in cancer cells. The results of FCM showed that down-regulation of miR-27a increased ADR accumulation and retention and decreased ADR releasing index, indicating that miR-27a had a direct or indirect function of pumping drug out of cells. The results of real-time PCR and western blot showed that miR-27a might mediate the expression of P-gp, which might function as an ATP-dependent drug-efflux pump.

## Conclusions

In conclusion, down-regulation of miR-27a might inhibit proliferation and drug resistance of gastric cancer cells through regulation of P-gp, cyclin D1 and p21. MiR-27a might be considered as a valuable target for cancer therapy.

## Competing interests

There is no conflict of interest. The authors declare that they have no competing interests.

## Authors' contributions

ZX and YL have made substantial contributions to conception and design, acquisition of data, and writing the manuscript. HJ participated in its design and gave final approval of the version to be published. All authors read and approved the final manuscript.
